# Multiseed liposomal drug delivery system using micelle gradient as driving force to improve amphiphilic drug retention and its anti-tumor efficacy

**DOI:** 10.1080/10717544.2018.1440669

**Published:** 2018-03-01

**Authors:** Wenli Zhang, Caibin Li, Ya Jin, Xinyue Liu, Zhiyu Wang, John P. Shaw, Bruce C. Baguley, Zimei Wu, Jianping Liu

**Affiliations:** a Department of Pharmaceutics, China Pharmaceutical University, Nanjing, PR China;; b School of Pharmacy, University of Auckland, Auckland, New Zealand;; c Auckland Cancer Society Cancer Research Centre, University of Auckland, Auckland, New Zealand

**Keywords:** Multiseed liposomes, active loading, micelle gradient, drug retention, efficacy

## Abstract

To improve drug retention in carriers for amphiphilic asulacrine (ASL), a novel active loading method using micelle gradient was developed to fabricate the ASL-loaded multiseed liposomes (ASL-ML). The empty ML were prepared by hydrating a thin film with empty micelles. Then the micelles in liposomal compartment acting as ‘micelle pool’ drove the drug to be loaded after the outer micelles were removed. Some reasoning studies including critical micelle concentration (CMC) determination, influencing factors tests on entrapment efficiency (EE), structure visualization, and drug release were carried out to explore the mechanism of active loading, ASL location, and the structure of ASL-ML. Comparisons were made between pre-loading and active loading method. Finally, the extended drug retention capacity of ML was evaluated through pharmacokinetic, drug tissue irritancy, and *in vivo* anti-tumor activity studies. Comprehensive results from fluorescent and transmission electron microscope (TEM) observation, encapsulation efficiency (EE) comparison, and release studies demonstrated the formation of ML-shell structure for ASL-ML without inter-carrier fusion. The location of drug mainly in inner micelles as well as the superiority of post-loading to the pre-loading method , in which drug in micelles shifted onto the bilayer membrane was an additional positive of this delivery system. It was observed that the drug amphiphilicity and interaction of micelles with drug were the two prerequisites for this active loading method. The extended retention capacity of ML has been verified through the prolonged half-life, reduced paw-lick responses in rats, and enhanced tumor inhibition in model mice. In conclusion, ASL-ML prepared by active loading method can effectively load drug into micelles with expected structure and improve drug retention.

## Introduction

Asulacrine (ASL), an inhibitor of topoisomerase II, has shown potent effect on breast and lung cancers (Baguley et al., 1984; Sklarin et al., 1992) which is mediated through the formation of DNA protein cross-links and DNA breakage (Covey et al., 1988; Baguley, 1990). However, phlebitis was occurred following intravenous (i.v.) infusion of ASL, which hampered its further development. ASL was administered intravenously as an isethionate salt, which is lipophilic (log P value ≈ 3.0 at pH 7) and moderately soluble in water (the solubility is ∼1 mg/mL, pH is ∼4.5; See et al., 2014). Our previous studies showed that post-injection drug precipitation upon dilution of body fluid and drug irritancy were probably the main reasons for phlebitis with ASL (See et al., 2014; Zhang et al., 2015a). Different strategies using liposome carriers have been attempted to improve phlebitis caused by ASL (Zhang et al., 2015a,b, 2016). However, the *in vivo* half-life of ASL in rabbits was still unsatisfactory and definitely shorter than that of carriers, suggesting insufficient drug retention in liposomes. Hence, there is considerable interest in the development of new formulation to improve drug retention for the prevention of phlebitis.

Recently, the multi-scale drug delivery system with tiny nanoparticles as ‘seed’ (gold particle, silica particle, or polymer nanoparticles such as pluronic, polystyrene, or PLGA (Yuk et al., 2011; Kim et al., 2013; Oh et al., 2013)) in the aqueous phase of larger liposomes/vesicles or inside a matrix nanoparticles (Wong et al., 2011), has gained significant attention, which could ‘bomb’ upon tumor microenvironment to meet different requirements from circulation and tumor penetration by size adaptation (Sunoqrot et al., 2014). Apart from the smart adapting function, this composite drug delivery system was hypothesized to improve drug retention by providing double barriers for drug release (Yuk et al., 2011). Hybrid micelles, self-assembled by two or more copolymers or surfactants, have been employed as promising drug carriers with the advantages of improved stability, potential drug-polymer interaction with more functional groups, small size, high drug loading capacity (Mikhail & Allen, 2009; Deng et al., 2012), and controllable drug release. Moreover, their hydrophilic surface and favorable size could benefit tumor penetration (Greish et al., 2004). Hence, hybrid micelles are suitable to be designed as ‘seeds’ of composite carriers. For the primary carrier, liposomes possess good biocompatibility against phlebitis (Liu et al., 2013), and timely burst release of ‘seed’ carriers for potentially-used tumor penetration (Reimhult, 2014) in the future. Therefore, the composite carriers with hybrid micelles encapsulated inside liposomes will be developed for the first time to improve phlebitis resulted from ASL leakage by controlling drug release.

For multiseed(ML) liposome system, there are three common preparation methods: passive loading method (Xin et al., 2017), fusion method with (Oh et al., 2013), or without electrostatic interaction (Yuk et al., 2011). For the passive loading method, multiseed liposomes are obtained by hydrating thin film with preformed drug-loaded seed carrier suspensions. For the fusion method, the drug-loaded seed carriers and empty liposomes were fabricated respectively and then mixed together for fusion. However, the final ML liposomes prepared by these two methods have very low drug encapsulation efficiency (EE) with most of drug lost in the external aqueous phase (Xu et al., 2012). To enhance EE, lipid membrane and seed carriers with opposite surface charges were employed (Siiman et al., 2009; Kim et al., 2013; Oh et al., 2013). Usually, the outer lipid membrane with negative charge (ξ < − 10 mV) is adopted in case of aggregation with serum protein or clearance by reticuloendothelial system (Liu et al., 2005; Miller et al., 2013). Accordingly, the charge of seed carriers should be positive in order to promote the fusion. However, the positive carrier is not feasible to load positive-charged weak bases like ASL. Therefore, how to fabricate composite sustained-release drug delivery system with high EE would be one of the problems that needs to be solved in this study.

In addition to unsatisfied EE, the conventional preparation method may bring about some other issues. Since the outer and inner carriers are both self-assembled soft matters, there exist risk for them to merge into mono-carrier (Banno et al., 2010) instead of forming multiseed-shell structure as expected. Furthermore, the drug loaded in seed carriers could also be loaded in the liposomal membrane. Therefore, drugs may shift to the outer membrane during carrier combination if it was loaded before. Unfortunately, only few reported studies focused on these questions which would be helpful to understand the distinct properties of the composite carriers and deserve further investigation.

In this article, we focused mainly on to improve ASL retention in carriers and also constituted a structure-verified composite carrier with high EE, hence an active loading method using micelle gradient was proposed. During this loading process, micelles inside the liposome acted as a pool to drive amphiphilic ASL traversing the lipid membrane into the aqueous phase of liposomes further before entering the micelles. To retain drug inside micelles and drive more drug to be loaded, a good retention property of drug-loaded micelles was a prerequisite. Therefore, the interaction of drug with micelles was studied to explain the mechanism of this method. Importantly, some reasoning experiments were conducted, that included influencing factor tests on EE, fluorescence and transmission electron microscope (TEM) observation, and *in vitro* release test, to verify the formation of composite carriers. Also, comparisons were made between commonly-used pre-loading and the active loading method developed here. The formulation optimization of ASL-ML was investigated to maximize EE and achieve satisfactory drug retention. Finally, the drug retention capacity of ML was evaluated through pharmacokinetic, drug tissue irritancy, and *in vivo* drug efficacy studies.

## Materials and methods

### Materials

Asulacrine (99% pure) was synthesized by Auckland Cancer Society Research Center, Auckland, Newzealand. The phospholipids, N-(carbonyl-methoxypolyethyleneglycol 2000)-1, 2-distearoyl-snglycero-3-phosphoethanolamine (DSPE-mPEG_2000_), dipalmitoyl-phosphatidylglycerol (DPPG), and hydrogenated soybean phospholipids (HSPC) were purchased from Lipoid GmbH (Ludwigshafen, Germany). Cholesterol was obtained from Sigma-Aldrich St. Louis, MO. D-α-Tocopheryl polyethylene glycol 1000 succinate (TPGS) were purchased from BASF SE Ltd. (Germany). Sodium Cholate (NaC) was provided from Meryer Chemical Technology Co., Ltd. (Shanghai, China). Acetonitrile was of chromatographic grade and all other reagents were of analytical grade.

### Animals

All the rats weighing between 0.18 and 0.22 kg were obtained from Qing Long Shan animal breeding grounds (Nanjing, China). The experiments were conducted in accordance with the National Act on Experimental Animals Guidelines (China) and have been approved by the University Animal Ethics Committee.

### Preparation of ASL formulations

***ASL-loaded hybrid micelles (ASL-M****)* ASL-M was prepared by a post-loading method. Firstly, the empty hybrid micelles (EM) were prepared by TPGS/DSPE-PEG (8:2 molar ratio). All the materials were dissolved in chloroform: methanol (3:1, v/v) and were later evaporated to form a thin lipid film in a round-bottom flask under vacuum using the rotary evaporator RE-85Z (Ying Yu Yu Hua Instrument Co., Gongyi, China). The film was further vacuum-dried overnight inside a desiccator and were then hydrated with a dextrose solution (5%, w/v) at 45 °C for 40 min. The resulting hybrid micelles were then filtered through 0.2 μm filters to obtain micelles with uniform size. To obtain ASL-M, the dispersions of preformed EM were incubated with an aqueous solution of ASL (1 mg/mL) at 37 °C for 1.5 h, the drug and carriers were at a mass ratio of 1:12.

***ASL-loaded multiseed liposomes (ASL-ML)*** To achieve satisfactory drug retention, post-loading (active loading method using micelle-gradient), and pre-loading methods were compared to prepare ASL-ML. The detailed methods is presented below.

For the post-loading method, phospholipid (DPPG or HSPC), DSPE-PEG_2000_ and cholesterol (mole ratio =11:1:9) were dissolved in chloroform: methanol (3:1, v/v) and were evaporated to form a thin film in a round-bottom flask under vacuum condition using a rotatory evaporator. The thin film was then hydrated with EM suspension (12 mg/mL suspended in 5% dextrose) prepared before and the empty ML was obtained. Thereafter, the ML suspension was extruded through 0.2 μm Nuclepore Track-Etch membranes (Whatman, UK) with an extruder (AE0001, Avestin Inc., Canada). An ultracentrifuge at 188,000 × g at 4 °C for 1 h was carried out to isolate the ML from the supernatant containing the un-encapsulated micelles. Then the ML pellets were incubated with an aqueous solution of ASL (1 mg/mL) at 37 °C for 1.5 h followed by a low speed centrifuge at 700 × g for 10 min to remove the unentrapped drug precipitate if any. Then an ultracentrifuge at 188,000 × g at 4 °C for 1 h was carried out to isolate the ASL-ML from soluble free ASL. The ASL-ML pellets were re-suspended using a dextrose solution (5%, w/v) and were kept at 4 °C in the dark.

With the pre-loading method, the lipid film was hydrated with ASL-M solution and the drug incubation process with ML was omitted. The other specific procedures were the same with those described in the post-loading method.

#### ASL-loaded liposomes (ASL-L)

To verify the role of micelle gradient in composite carriers, ASL-L were also prepared using post-loading method. For the ASL-L, only the hydration medium for the lipid film was replaced by dextrose solution(5% m/v) without micelles. The other operation processes including drug loading were the same.

### Formulation optimization of ASL-ML

To maximize EE of ASL-ML and enhance drug retention by post-loading method, various factors were investigated that included hydration media, type of phospholipids, and EM concentration. Meantime, the ML-shell structure could be indirectly confirmed by EM concentration effect on EE.

#### Hydration medium

The EM dispersion (12 mg/mL) prepared with various hydration media (5% dextrose solution, 250 mM ammonium sulfate, and 0.12 M PBS) was used to hydrate the lipid thin film prepared beforehand. There were no significant differences between these EMs in particle size and stability. With the ammonium sulfate medium, a trans-membrane ion gradient was generated by removing the unentrapped ammonium sulfate using dialysis (Zhang et al., 2016). The other procedures were same as those for ASL-ML described above. The size, EE, and drug loading (DL) of ASL-ML prepared with different hydration media were investigated to determine the optimal hydration medium for the following experiments.

#### Type of phospholipids and drug loading temperature

Two types of phospholipid (DPPG and HSPC) with different phase inversion temperatures (PIT) and charges were selected to prepare lipid thin film. Accordingly, different incubation temperature (37 °C and 41 °C for DPPG, 37 °C and 55 °C for HSPC) were investigated to investigate the effect of membrane fluidity at different temperatures on EE. PIT (41 °C for DPPG, 55 °C for HSPC) was chosen to facilitate the formation of thin film and body temperature was chosen to predict the drug leakage behavior from carriers *in vivo*. The other procedures were the same as those described for preparation of ASL-ML. The size, EE, and DL of ASL-ML prepared with different phospholipids (ASL-ML (HSPC) or ASL-ML (DPPG)) at different loading temperatures were investigated to determine the optimal formula for the following experiments.

#### EM concentration

To verify the role of micelles for drug loading in ASL-ML and to optimize the number of micelles in the aqueous core of liposome, the prepared liposome film was hydrated with the same volume of medium without or with EM of different concentrations (9, 12, 18, and 60 mg/mL, respectively ). ASL was loaded at a temperature optimized for each type of phospholipid. The other procedures were the same as those described for preparation of ASL-ML. The appropriate concentration of EM was determined by the size, EE, and DL of ASL-ML.

### The critical micelle concentration (CMC) determination of blank micelles and ASL-M

To investigate the interaction between drug and micelle carriers, the CMC of EM is compared with that of ASL-M using drop weight method (Permprasert & Devahastin, 2005; Ravindra, 2007). Firstly, the optimized EM and ASL-M were prepared with same formula and method which were later diluted to a series of concentrations from 0.002 to 0.25 g/L (total polymer mass concentration) with deionized water, respectively. Then the solution was added to a vertically-fixed acid biuret to drip and 30 drops of the micelle solution were weighed. The average mass of per drop was calculated and plotted against the concentration of polymers to obtain CMC from the intersection value of two extended regression lines.

### *In vitro* drug release from ASL-M, ASL-L, and ASL-ML

#### *In vitro* drug release in phosphate buffered saline (PBS)

Drug release behaviors from ASL-M, ASL-L (DPPG), ASL-L (HSPC), ASL-ML (DPPG), and ASL-ML (HSPC) all prepared with post-loading method were compared with ASL solution (1 mg/mL in 5% dextrose) as control using dialysis method as reported previously (Zhang et al., 2016). At pre-determined time points (1, 3, 6, 12, 20, and 24 h), samples (100 μL) were withdrawn from the dialysis bags and were centrifuged at a low speed at 700 × g for 10 min to remove the drug precipitate. The amount of free drug diffused from the dialysis bag was ignored since it had a capacity to quickly diffuse out (>80% in 20 min) and was 500 times diluted. The unreleased ASL in formulations was extracted by dissolving in acetonitrile before high-performance liquid chromatography (HPLC) analysis. More stable ASL-ML was chosen for the later release study in serum.

#### *In vitro* drug release in serum

The optimized ASL-ML suspension was mixed with an equal volume of rabbit serum in a tube, which was placed in a shaking water bath at 37 °C for 1 h. Then the mixture of ASL-ML and serum was ultracentrifuged at 188,000 × g at 4 °C for 1 h to isolate the released drug from ASL-ML. ASL-ML pellets were collected and re-suspended using a dextrose solution (5%, w/v). Then ASL in ASL-ML adsorbed with serum proteins was extracted by protein precipitation method using acetonitrile before HPLC analysis, similar to the processing procedures used for the blood samples.

### Characterization of ASL-M, ASL-L, and ASL-ML

Size and zeta potential of ASL-M, ASL-L, and ASL-ML were measured with dynamic light scattering (DLS) using a Malvern Nano ZS (Malvern Instruments, UK). All measurements were conducted at 25 °C in triplicate.

The drug concentration in all initial samples was the total drug concentration (*C*_0_). To determine the EE and DL of ASL-M, dialysis method was used to remove the free drug (*C*_f1_). The encapsulated drug in ASL-M was calculated according to Equation (1). For ASL-L or ASL-ML, the potential drug precipitate outside ASL-L or ASL-ML was firstly removed using low speed centrifuge. Then the supernatant was collected (C*_1_*) and further ultracentrifuged to separate the free drug (*C*_f2_) from ASL-L or ASL-ML suspension. The encapsulated drug in ASL-L or ASL-ML was calculated according to Equation (2). The *C*_1_, *C*_0_, and *C*_f_ were determined by HPLC after extracting the ASL with acetonitrile to calculate the EE and DL for ASL-M, ASL-L, and ASL-ML using Equations (3) and (4):

(1) $$M_{\text{drug}}^{\text{′}}\;=\;(C_{0}\;–\;C_{\text{f1}})\text{ × }V_{\text{total}}$$

(2) $$M_{\text{drug}}^{\text{′}}\;=\;(C_{1}\;–\;C_{\text{f2}})\text{ × }V_{\text{total}}$$

(3) $$\text{EE}\left(\%\right)=\frac{M_{\text{drug}}^{\text{′}}}{M_{\text{drug}}}\text{ × }\text{1}00$$

(4) $$\text{DL}\left(\%\right)\;=\;\frac{M_{\text{drug}}^{\text{′}}}{M_{\text{lipids}}\;+\;M_{\text{drug}}}\text{ × 1}00$$

where *V*_total_ is the total volume of original ASL-M, ASL-L, or ASL-ML suspension, *M*′_drug_ is the mass of encapsulated drug in ASL-M (Equation (1)), ASL-L, or ASL-ML suspension (Equation (2)), *M*_drug_ and *M*_lipids_ are the mass of the total drug and carrier materials used in the ASL-M, ASL-L, or ASL-ML, respectively. The concentration of drug was analyzed using a validated HPLC method (Zhang et al., 2016).

The morphology of ASL-M, ASL-L, and ASL-ML was observed by TEM (Hitachi Ltd., Japan). Briefly, samples were diluted appropriately and dropped on a copper grid and then stained with uranyl acetate (2%, w/v, pH 6.8), followed by air drying before TEM observation.

### Structure certification by fluorescence imaging in giant particles

In order to be visualized under microscope, all the carriers were prepared in micrometer magnitude without extrusion or filtration and labeled by fluorescent dyes. Firstly, dual fluorescence-labeled ML (d-ML) with DSPE-PEG_2000_-Fluorescein isothiocyanate (FITC) and Nile red was prepared to confirm the ML-shell structure of ML. In d-ML, DSPE-PEG_2000_-FITC was employed to assemble micelles. Lipophilic Nile red was added during thin film formation to label bilayer membranes. The other preparation procedures of d-ML were the same with those of ASL-ML prepared by post-loading method. To further investigate the effect of different drug loading methods on drug location, Nile red-labeled ML with pre-loaded FITC (n-ML-pre) and Nile red-labeled ML with post-loaded FITC (n-ML-post) were prepared. Here, Nile red was also used to visualize the liposomal membrane and free FITC was employed as a marker to substitute drug due to their similar amphiphilic properties (logP for ASL and FITC is 3.8 and 3.6, respectively). The preparation methods were the same with those described for preparation of ASL-ML and FITC was loaded in a similar pre- or post-loading way to drug. Then, these three kinds of fluorescence-labeled ML were observed by confocal laser scanning microscope (CLSM; LSM700, Carl Zeiss, Germany).

### Pharmacokinetics in rats

Twenty-four male Sprague-Dawley rats (180 ± 20 g) were randomly divided into four groups and were administrated with ASL solution, ASL-M, ASL-L, and ASL-ML at a dose of 10 mg/kg via tail vein injection. The formulations were diluted with 5% (m/v) dextrose solution to give a final ASL concentration of 1 mg/mL and were sterilized by filtration through a 0.22 μm membrane. Following the completion of drug administration, about 200 μL of blood samples were collected from the orbital plexus at the selected intervals (0, 0.25, 0.5, 1, 2, 4, 6, 8, and 12 h) and centrifuged at 700 × g for 10 min to obtain the plasma samples. For drug analysis, ASL was extracted using protein precipitation method (Zhang et al., 2016). The drug concentration was linear in the range of 0.1–5 μg/mL (*r* > 0.99) with no interference from the plasma. Pharmacokinetic parameters were calculated using the non-compartment analysis (WinNonlin; professional edition, version 3.1; Pharsight Co., Mountain View, CA).

### Drug irritancy using rat paw lick/lift test

Thirty rats were randomly divided into five groups (*n* = 6), Later each rat received a single injection of 0.1 mL of each test formulation (ASL-solution, ASL-L, ASL-M, and ASL-ML) and dextrose solution (5%, w/v) as negative control under plantar into the left paw. The formulations all contained ASL of 1 mg/mL after dilution with dextrose solution (5%, w/v). The paw-lick/lift responses were monitored over a period of 20 min. Additionally, to detect responses that might be temporally delayed, clinical signs of redness, or swelling at the injection site were recorded after dosing post 48 h.

### *In vivo* anti-tumor activity

For *in vivo* implantation, female BALB/c mice were subcutaneously injected in the right flank with 0.1 ml of cell suspension containing 1 × 10^7^/mL mouse 4T1 breast tumor model cells in serum-free culture medium. The *in vivo* anti-tumor studies were started when the tumor volumes reached about 100 mm^3^ (Day 0). Twenty-four 4T1 tumor-bearing mice were randomly divided into four groups (six mice per group): group 1 was treated with 5% dextrose solution and groups 2–4 were treated with 1 mg/mL ASL formulations (ASL dextrose solution tested in clinical trials, ASL-M, and ASL-LM;10 mg/kg), respectively. Mice received administration intravenously through the tail vein every other day for 14 days. Tumor volume and body weight were monitored before every injection during 14 days. The tumor volume (V) was calculated by the equation V = (*a* ×* b*^2^)/2, where *a* represents the longest diameter and *b* represents the shortest diameter vertical to length. At the end of the experiment, all the animals were sacrificed and the tumor tissues were harvested and weighed. The tumor growth inhibition was calculated by (*C* − *T*)/*C* × 100%, where *T* represents the average tumor weight after treatment and C represents the average tumor weight of the control group.

### Statistical analysis

Data were expressed as mean ± standard deviation. Statistically significant differences were determined by two tailed Student's t-test using GraphPad Prism 5 software (GraphPad Software Inc., San Diego, CA) with *p* < .05 as a level of significance.

## Results

### Optimization of ASL-ML preparation

ASL-M was observed as a monomodal size distribution with an average size of 24.3 ± 1.5 nm, small enough as the seed carriers. The EE of ASL-M prepared with post-loading method was 89.20 ± 2.50%, which predicted that the drug might be well loaded post the formation of composite drug carriers. To optimize the drug loading and retention in ASL-ML, various conditions were compared. Table 1 shows that the different formulations exhibited much discrepancy in EE and DL. All the size of ASL-ML formulations were around 170–200 nm with ASL-ML (DPPG) smaller than ASL-ML (HSPC).

**Table 1.** Effects of different parameters on size distribution, polydispersity index, entrapment efficiency, and drug loading of ASL-ML (mean ± SD, *n* = 3).

Conditions	Parameters	Size (nm)	PDI	EE (%)	DL (%)
Loading methods	Pre-loading (DPPG)	178.6 ± 2.3	0.192 ± 0.025	3.40 ± 0.41	0.16 ± 0.02
	Post-loading (DPPG)	172.2 ± 2.4	0.216 ± 0.027	78.02 ± 2.62*	3.61 ± 0.13
	Pre-loading (HSPC)	190.6 ± 2.3	0.216 ± 0.028	3.31 ± 0.09	0.16 ± 0.01
	Post-loading (HSPC)	193.7 ± 1.7	0.220 ± 0.023	31.31 ± 1.62*	1.48 ± 0.08
	5% dextrose with EM	172.2 ± 2.4	0.216 ± 0.027	78.02 ± 2.62*	3.61 ± 0.13
Hydration media^a^	Ammonium sulfate with EM	172.0 ± 2.5	0.209 ± 0.025	56.81 ± 2.13	2.66 ± 0.10
	PBS with EM	167.8 ± 1.9	0.203 ± 0.024	41.04 ± 2.51	1.93 ± 0.12
Type of phospholipid and incubation temperature	DPPG (37 °C)	172.2 ± 2.4	0.216 ± 0.027	78.02 ± 2.62	3.61 ± 0.13
	DPPG (41 °C)	175.9 ± 2.6	0.208 ± 0.020	81.22 ± 2.13	3.76 ± 0.09
	HSPC (37 °C)	193.7 ± 1.7	0.220 ± 0.023	31.31 ± 1.62	1.48 ± 0.06
	HSPC (55 °C)	192.3 ± 1.5	0.117 ± 0.025	40.67 ± 1.42*	1.91 ± 0.06
EM concentration (mg/mL)	0^b^ (DPPG)	177.1 ± 3.1	0.219 ± 0.029	39.01 ± 0.92	1.91 ± 0.05
	12 (DPPG)	175.9 ± 2.6	0.208 ± 0.020	81.22 ± 2.13*	3.76 ± 0.10
	18 (DPPG)	193.7 ± 1.8	0.104 ± 0.028	81.41 ± 2.23	3.69 ± 0.10
	0^b^ (HSPC)	192.6 ± 2.4	0.198 ± 0.028	10.73 ± 0.61	0.53 ± 0.03
	12 (HSPC)	194.6 ± 1.8	0.119 ± 0.020	40.32 ± 1.21	1.90 ± 0.06
	18 (HSPC)	192.5 ± 1.6	0.113 ± 0.019	42.63 ± 1.33	1.97 ± 0.06
	60^c^ (HSPC)	205.8 ± 1.7	0.105 ± 0.018	51.72 ± 1.52*	2.10 ± 0.06

a The typical data of ASL-ML prepared with DPPG, since there were no significant differences in size, EE, and DL between ASL-ML prepared with DPPG and HSPC.

b ASL-ML without inner micelles which could also be termed as ASL-L.

c The upper limit of EM concentration which could not induce micelle aggregation.

* Significant differences from other formulations in the same condition group.

#### Drug loading methods

For the pre-loading method, the EE of ASL was less than 4% corresponded to the ratio of internal aqueous phase to the total volume of liposomes (Xu et al., 2012). The final EE and DL of ASL-ML (DPPG) and ASL-ML (HSPC) by post-loading method was much higher (Table 1). Therefore, the post-loading method was feasible to load ASL and were further optimized in later studies.

#### Hydration medium

It was obvious that the maximized EE was achieved when 5% dextrose with EM were employed. In contrast, PBS gave rise to the lowest EE which may be ascribed to the reduced drug solubility in PBS. However, the EE of ASL-ML was still much higher than that of ASL-L using PBS as hydration medium (data is not shown, less than 3% in our preliminary study), which also proved the effect of micelles in drug loading. As for ammonium sulfate gradient, this method was not as effective as that for ASL-L reported in our previous study (Zhang et al., 2015a). This could be explained by the fact that the acid intra-liposomal pH of ASL-ML which was not as low as that for ASL-L due to the presence of micelles drove very limited drug entering the liposomes and made further encapsulation of ionized drug by micelles difficult (Lee et al., 1998).

#### Type of phospholipids and drug loading temperature

As seen from Table 1, ASL-ML (DPPG) had a higher EE than ASL-ML (HSPC) and temperature only contributed slightly to EE increase for ASL-ML (DPPG) (*p* > .05), indicating that DPPG with negative charge was helpful to load positive-charged drug. However, the EE of ASL-ML (HSPC) increased significantly only when the temperature was beyond PIT of HSPC, which may predict better drug retention effect when using HSPC. Based on the EE and DL, the optimal temperature for drug loading was set at 37 °C for ASL-ML (DPPG) and 55 °C for ASL-ML (HSPC) in later studies.

#### EM concentration

For ASL-ML (DPPG), the EE doubled with 12 mg/mL of EM compared to ASL-L without inner EM (0 mg/mL) but no more improvement was noted with higher EM concentration. Hence 12 mg/mL of EM was employed to prepare ASL-ML (DPPG). Similarly, the EE of ASL-ML (HSPC) prepared with 12 mg/mL of EM increased almost three folds with aid of inner EM and their EE increased even more with higher EM concentration and achieved the maximum with at a concentration of 60 mg/mL of EM. Since the number of encapsulated micelles was proportional to the concentration of EM to some extent (Banno et al., 2010), higher concentration of EM in aqueous phase (Siiman et al., 2009) would involve more encapsulated EM and definitely enhance the drug loading capacity. Therefore, the larger ASL-L (HSPC) which could accommodate more micelles than ASL-L (DPPG) achieved saturation at a higher EM concentration. However, the micelles with concentration above 60 mg/mL easily led to aggregation. Therefore, an upper limit of 60 mg/mL was chosen to be the optimum EM concentration for ASL-ML (HSPC).

### The interaction of drug and micelles determined by CMC

**Figure 1.**
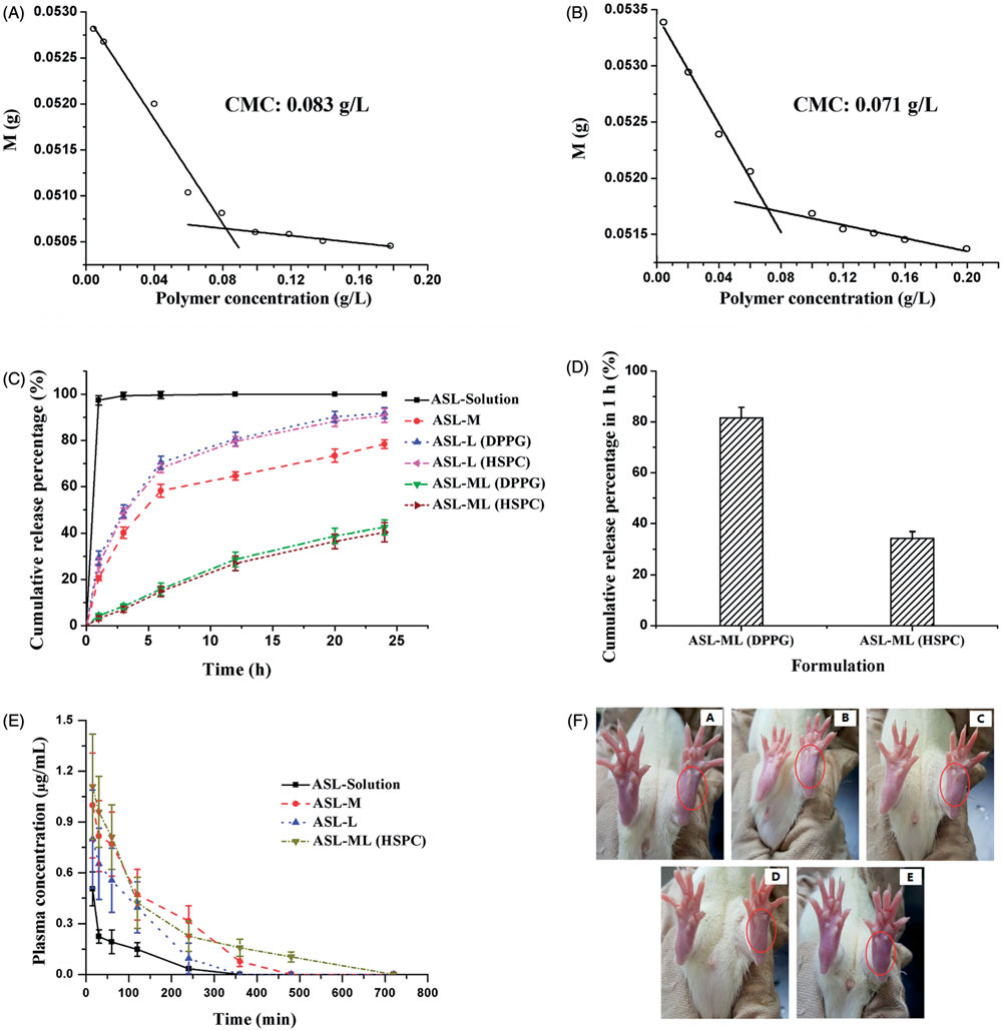
CMCs of empty micelles (A) and ASL-loaded DSPE-PEG/TPGS micelles (B) (mean ± SD, *n* = 3) determined by drop weight methods. Percentage of cumulative drug release from different ASL formulations in pH 7.4 PBS (C) and rabbit serum (D) at 37 °C (mean ± SD, *n* = 3). (E) Plasma concentration-time curves of ASL in rats after i.v. injection of ASL-Solution, ASL-M, ASL-L, and ASL-ML (HSPC) at a dose of 10 mg/kg (mean ± SD, *n* = 6). (F) Typical rat paws (in the circles) at 24 h after a subplantar injection of ASL solution (a), ASL-L (b), ASL-M (c), ASL-ML (d), and 5% dextrose solution (e). The right paws without receiving any injection were employed as self-control.

Since the micelle gradient would be utilized to load drug in the composite carriers, it is necessary to investigate whether the interaction between drug and micelle carriers exist to serve as a locker for potential drug driving similar to other active loading method (Gubernator, 2011). After ASL was loaded in the micelles with DSPE-mPEG_2000_ and TPGS, the CMC value was reduced from 0.083 to 0.071 g/L (Figure 1(A,B)). Therefore, there might be an interaction between drug and micelles. Also, it was inferred that the tendency of drug diffusion into micelles might be larger than out of micelles, which could well explain the mechanism of this micelle-gradient drug loading method.

### *In vitro* PBS and serum release

As shown in Figure 1(C), ASL encapsulated in both liposomes (DPPG and HSPC) exhibited certain controlled release effect compared to ASL solution and no significant differences was noted between them. Interestingly, ASL-M could slow down the drug release more obviously than ASL-L possibly due to the interaction of micelles and drug. However, the burst release was still not arrested with almost 40% drug released in the first 3 h. Benefited from two combined carriers, ASL-ML exhibited the most satisfied drug release and the accumulative release percentage in 24 h was only ∼40% and the release profiles of ASL-ML prepared with DPPG and HSPC also showed no difference in PBS. Surprisingly, the drug retention capacity was differentiated by changing the release medium to rabbit serum. Seen from Figure 1(D), there was a rapid drug efflux observed at 1 h for ASL-ML (DPPG). However, ASL-ML (HSPC) was still robust to retain more than 60% drug in carriers and hence was selected to carry on in the later studies. Although drug release in serum in 1 h is not low enough, it was still acceptable for an amphiphilic drug in liposomal system (Boman et al., 1993). The different results obtained from PBS and serum medium also highlighted the importance of a bio-relevant release method.

### Transmission electron microscopy

**Figure 2.**
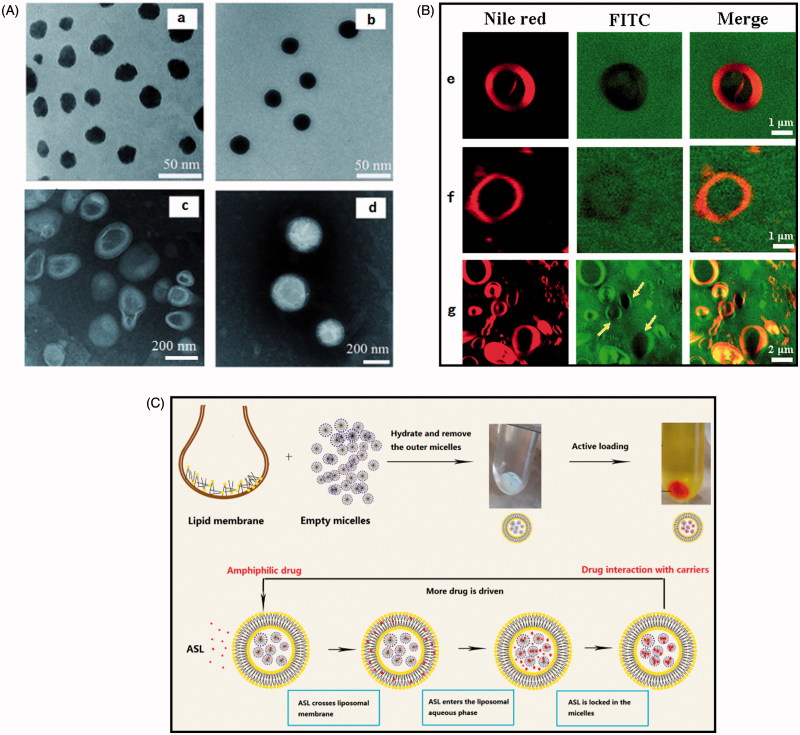
(A) TEM micrographs of ASL-M (a,b), ASL-L (c) and ASL-ML (HSPC) (d). The micelle lipid concentration (m/v) for a and b were 12 and 60 mg/mL, respectively. (B) Giant particles labeled with Nile red, FITC, or DSPE-PEG2000-FITC observed by confocal laser scanning microscope. d-ML (e), n-ML-post (f), and n-ML-pre (g). (C)Preparation of ASL-ML by active loading method using micelle gradient and the possible mechanism for this method.

The morphology of micelles prepared with polymer concentration of 12 and 60 mg/mL, optimized ASL-L (HSPC) and ASL-ML (HSPC) was analyzed by TEM. As shown in Figure 2(A), both micelles were spherical with a homogenous size of around 20 nm, which was in accordance with the size determined by DLS. A hydration layer could be observed on the shell of micelles. Interestingly, the edge of micelles prepared with low polymer concentration was rough and the density of micelles under microscopy was uneven. By increasing the concentration of polymers, micelles became perfectly round and smooth with homogenous density. Compared to ASL-ML, ASL-L exhibited hollow structure of vesicle with fingerprint, which belongs to typical liposomes. ASL-ML were more like solid nanoparticles and with patches on it, suggesting a ML structure different from ASL-L. Unfortunately, the shell of ASL-ML cannot be observed clearly under the current resolution of TEM.

### Structure certification by fluorescence imaging in giant particles

From Figure 2(B), three kinds of ML with an obvious vesicle structure judging from the Nile red circle could be observed. Since the unentrapped micelles cannot be removed by centrifugation due to the relatively large size, the green fluorescence was observed both inside and outside liposomes. Although the micelles were not discernible under microscope, some evidence for structure certification could be found. When micelles were labeled by DSPE-PEG_2000_-FITC (d-ML), green fluorescence was observed inside and outside vesicles except for the vesicle membrane, showing a black circle overlapped with Nile red (Figure 2(B-e)), which proved that the micelle did not adsorbed on or inserted in the vesicle membrane. In addition to the overlapped black circle with Nile red, the vesicle structure also confirmed this hypothesis. The explanation was that the liposomes would change into solid sphere (Edwards et al., 1997) if the high concentration of DSPE-PEG_2000_-FITC were inserted into membrane. When FITC was loaded post the formation of ML (n-ML-post), the phenomenon was similar to that for d-ML. Moreover, the intensity of green fluorescence inside and outside liposomes was similar. Judging from the overlapped black circle with Nile red and close intensity of green fluorescence inside and outside vesicles, it was inferred that FITC could diffuse into micelles instead of being arrested by membrane. Interestingly, when FITC was pre-loaded in the micelles (n-ML-pre), the FITC shifted from micelles into the liposomal membrane and FITC completely merged with Nile red after the formation of ML and the green fluorescence intensity inside most of the vesicles was less than that outside (yellow arrows in Figure 2(B-g)), suggesting FITC leakage from micelles using pre-loading method.

### Pharmacokinetics in rats

Figure 1(E) depicts the pharmacokinetic profiles of ASL-L, ASL-M, and ASL-ML in comparison with ASL solution following i.v. injection in rats. Although ASL-ML, ASL-L, and ASL-M all showed higher onset concentrations and larger areas under the curve (AUC; *p* > .05) than ASL solution, there were no significant differences in elimination half-lives between ASL-L and ASL solution (Table 2), suggesting drug leakage from ASL-L (Zhang et al., 2015 b). However, MRT of ASL-L was much higher than that of ASL solution, indicating that liposome carriers may influence the distribution of ASL encapsulated and ASL-ML exhibited the longest half-life about 2.8 times as long as ASL solution and twice as long as ASL-M. Since the size and surface properties of ASL-L and ASL-ML were similar, their different drug behaviors *in vivo* were probably ascribed to different drug retention capacities between mono- and composite carries. Also, the order of MRT coincided with the order of release rate in PBS for different ASL formulations, which proved that increased drug retention in PEGylated carriers could contribute to an increase in drug circulation time.

**Table 2.** Pharmacokinetic parameters of different ASL formulations following i.v. injection to rats at a dose of 10 mg/kg (means ± SD, *n* = 6).

Parameters	Units	ASL-solution	ASL-ML	ASL-M	ASL-L
T_1/2_	min	74.98 ± 13.67^▴^	211.7 ± 20.81*	102.99 ± 16.34*^▴^	75.14 ± 16.77^▴^
AUC	μg/ml*min	48.66 ± 2.08^▴^	202.97 ± 17.92*	169.83 ± 11.68*^▴^	110.31 ± 11.11*^▴^
MRT	min	84.25 ± 10.06^▴^	239.65 ± 41.55*	142.97 ± 18.46*^▴^	102.28 ± 22.15*^▴^
CL	(mg/kg)/(μg/ml)/min	0.205 ± 0.025^▴^	0.049 ± 0.005*	0.059 ± 0.007*	0.091 ± 0.006*

*p < .05 vs. ASL-Solution.

^▴^*p* < .05 vs. ASL-ML.

### Drug irritancy using rat paw lick/lift test

Table 3 shows the drug irritancy and highlighted the improvement of tissue compatibility of ASL-ML. In addition, the paws exposed to ASL solution were red and swollen at 48 h (Figure 1(F)). ASL-L and ASL-M caused response of paw lifting/licking and slight swelling but no obvious purple color was noted in the paw (Figure 1(F-b,c)). In contrast, injection of ASL-ML caused little animal response and no visible changes in the paw was noted when compared with the injection of dextrose solution (5%, w/v) (Figure 1(F-d,e)). Consistent with pharmacokinetic studies, ASL-ML showing the best drug retention behavior could prevent drug irritation more effectively.

**Table 3.** Rat paw lick/lift response to subplantar injection of 0.1 mL of ASL formulations with dextrose solution as negative control (*n* = 8).

		Paw-lick		Paw-lift		
Formulation	*n*	Percent of response^a^	Total number/rat^b^	Percent of response^a^	Total number/rat^b^	Percent of response at 48 h^c^
Dextrose solution	8	0	0	37.5	0.6	0
ASL-solution	8	100	9.0	100	15.6	25
ASL-M	8	50	2.7	50	1.7	0
ASL-L	8	62.5	3.8	87.5	2.8	0
ASL-ML	8	0	0	50	1.6	0

a The percent of rats showing response within 20 min.

b Total number of response within 20 min per rat.

c Percentage of rats that showed response upon touch.

### *In vivo* anti-tumor activity

**Figure 3.**
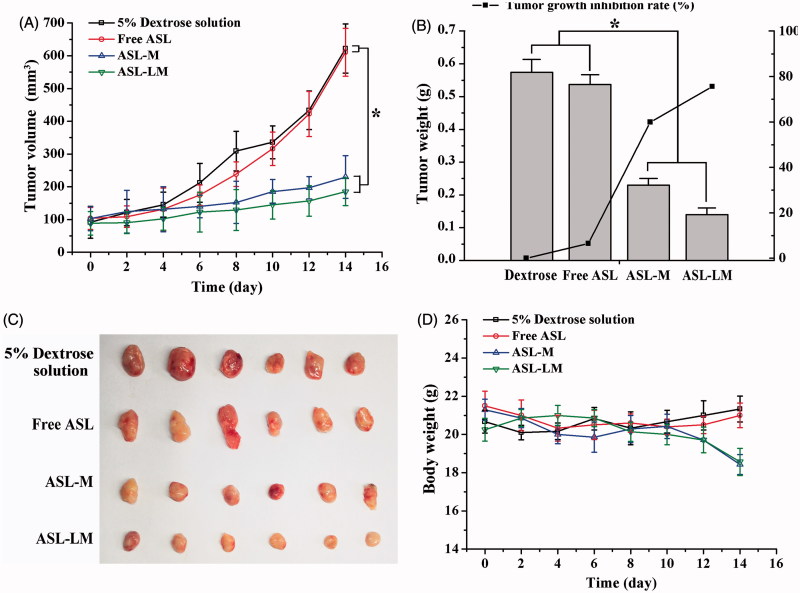
In vivo anti-tumor study of ASL formulations in BALB/c mice implanted with 4T1 cells (mean ± SD, *n* = 6). A.(A) Tumor volume was monitored every other day during administration. (B) Tumor weight was measured at the end of the experiment to calculate the tumor inhibition rate. (C) Image of 4T1 tumor tissues after treatment with 5% dextrose solution and different ASL formulations for 14 days. (D) Changes of body weight during administration.

As shown in Figure 3(A), the tumor growth in control and ASL solution groups was much more rapid than those in ASL-M and ASL-LM groups. The tumor growth and inhibition rate (Figure 3(B)) in ASL-solution group was quite similar to that in control group. These results were consistent with *in vivo* half-lives of ASL formulations. ASL-LM was found to be a little more effective at inhibiting the growth of tumor in comparison with ASL-M, but no significant differences (*p* > 0.05) were observed, which may be due to the better tumor penetration efficiency of ASL-M than ASL-LM, although ASL-LM has longer T_1/2_ and bigger AUC than ASL-M. The body weights of the mice in the ASL-M and ASL-LM group decreased a little, but no significant differences were observed, indicating good tolerance. The decreased body weight compared with ASL solution might be ascribed to the anti-tumor effect of ASL, since little drug was bioavailable during i.v. administration of ASL solution, leading to little drug systematic side effect.

## Discussion

Post-infusion drug precipitation may be one of the major reasons for phlebitis in clinical trials of ASL. Sufficient drug retention in the formulation and high EE are highly important to avoid *in vivo* drug precipitation and improve biocompatibility. To enhance the ASL retention in the formulation, composite micelles-contained liposomes (ML liposomes) with high EE was designed and optimized using a novel micelle-gradient method in this study. To be sure with the formation of composite carriers, some reasoning experiments about the mechanisms for micelles-gradient method was performed wherein the location of ASL in ASL-ML, the structure of ML had been carried out. Meantime, drug release, pharmacokinetic, drug tissue irritancy, and *in vivo* anti-tumor activity studies were carried out with control of ASL free solution, ASL-L, or ASL-M to evaluate the drug retention capacity of ASL-ML *in vitro* and *in vivo*. All these will be discussed below in detail.

In micelle gradient method (Figure 2(C)), the lipid film was hydrated with blank micelle solution to obtain blank ML, then the extra-liposomal micelles were removed by ultracentrifugation to generate a trans-membrane micelle gradient. After that, the empty ML pellets were incubated with drug solution, in which neutral ASL molecule could diffuse into inner aqueous phase of liposomes due to ASL trans-liposomal membrane concentration gradient. Then the ASL in liposomal aqueous compartment further diffused into the micelles, leading to a decreased ASL concentration inside liposomes. Hence, more extra-liposomal ASL can be driven into liposomes. The principle of micelles gradient method is similar to that of active loading method using metal gradient, in which metal ion served as a trapper to form poorly-soluble complexity with drug (Abraham et al., 2004). Here, the micelles could serve as a driving force of drug loading and meantime also acted a locker to retain ASL inside liposomes.

During this drug-loading process by micelle gradient, two preconditions are necessary. One is the amphiphilicity of drug, i.e. drug should be compatible in both hydrophobic and hydrophilic phase, since ASL was supposed to cross the lipidic liposomal membrane to the aqueous phase and then diffuse into the micelles. Reasoning for this loading theory was that the encapsulated ASL could have leaked out of micelles to the aqueous phase of liposomes and then to the outside. However, this did not happen. With the manipulation of the conditions of post-loading using micelle-gradient, a maximal EE of 81.2% and DL of 3.76% (w/w) were achieved for the ASL-ML (DPPG). To explain this paradox, the interaction between drug and micelles (the second precondition) is needed, which was proved by the lower CMC of drug-loaded micelles than that of blank micelles. It was reported that encapsulation of hydrophobic drug into the micelles would enhance the hydrophobic force, which could not only enhance the structural stability of micelles but also prevent drug leakage (Cammas et al., 1997). Since the encapsulation of ASL in micelles increased the micelles packing through the interaction of drug and materials, the drug entering micelles could be locked and could not diffuse reversely.

ML was supposed to have the structure of multiple micelles cores and liposome shell. However, in the process of hydration, DSPE-PEG used in both micelles and liposomes may cause inter-carrier adsorption or fusion due to the hydrophobicity of DSPE block (Banno et al., 2010). If this happened, the micelles inside were not able to form and the lipid-bilayer shell would gain more PEGylation, which might transform the vesicle to the spherical structure (Edwards et al., 1997) or impede drug loading due to the increased hydrophilicity and steric hindrance of membrane. However, fluorescent photos for giant particles showed the diminished green fluorescence of DSPE-PEG_2000_-FITC in liposome membrane, which ruled out the possibility of membrane fusion between micelles and liposomes. In addition, the final EE of ASL-ML (DPPG) is 81.22 ± 2.13%, which was only 39.01 ± 0.92% (Table 1) when using the dextrose solution (5% (m/v)) to hydrate the liposome film instead of the micelles solution (similar to the trend of EE with ASL-ML (HSPC)). The increased EE with the aid of micelles further proved the exclusion for membrane fusion.

Since ASL is amphiphilic, it might be loaded theoretically either in the liposomal membrane, or intra-liposomal aqueous apartment, or in micelles. To further confirm the location of ASL, influencing factor tests on EE and release experiments have been done. As shown in Table 1, the EE of ASL-ML (DPPG) (81.41 ± 2.23%) was two folds greater than that of ASL-L (DPPG) (39.01 ± 0.92%) with drug post-loaded by the same method, which proved that more drug could be loaded with the aid of micelles. This finding evokes the question that half percentage of drug in ASL-ML may exist in the liposomal membrane or aqueous phase. If this was true, drug released from ASL-ML should be beyond half of that released from ASL-L in theory. However, ASL-ML obviously slowed down the drug release compared with ASL-L by more than half which is shown in Figure 1(C). ASL-ML gave rise to a 1.8 times longer *in vivo* half-life than ASL-L (Table 2). Also, the fluorescent photo (Figure 2(B-f)) proved that FITC loaded in the same way with ASL was absent in the liposomal membranes. Furthermore, ASL could be post loaded in the micelles to form stable ASL-M with a high EE and a sustained release (Figure 1(C)), which provided a feasibility for ASL to traverse the lipid membrane and further diffuse into micelles. However, there must be a diffusion equilibrium between ASL in inner aqueous phase of liposomes and ASL in liposomal membrane and micelles, therefore our studies only suggested that most of drug instead of 100% was located in micelles.

Other than micelle-gradient method, passive loading method was also investigated. Compared to post-loading method using micelle gradient, there were two drawbacks for pre-loading method. Firstly, almost 96% of ASL-M in the external aqueous phase was wasted during thin film hydration, since the percentage of hydrophilic drug (here is drug-loaded micelles) entrapped corresponded to the volume ratio of internal to the total volume of liposomes (Xu et al., 2012). Even the same drug content may be achieved by increasing DL of pre-loaded micelles, the EE is still very low with most of drug lost in the external aqueous phase. Secondly, drug pre-loaded in micelles can shift from micelles to liposomal membrane during combination of two carriers as featured in Figure 2(B-g). When the thin film was hydrated at a temperature higher than PIT, the molecular packing of liposome membrane and micelles became loose, which facilitated drug transfer from micelles to liposome bilayers. By contrast, drug was always driven inwards when using post-loading method.

To evaluate the effectiveness of the ASL-ML composite carriers in drug retention improvement, drug release properties, pharmacokinetics, drug irritancy, and *in vivo* anti-tumor activity of ASL-ML were compared with the control groups that included ASL free solution, ASL-L, or ASL-M. The most sustained release patterns were observed for ASL-ML, which arrested the drug leakage when >60% drug was retained in the ASL-ML over a period of 24 h. This was due to the combined control of lipid bilayer of liposomes and the micelles on the release of ASL from carriers. Following i.v. injection to rats, the half-life of ASL-L was identical with that of free drug, suggesting drug leakage from liposomes. ASL-ML showed prolonged half-life than free drug and ASL-M. Moreover, ASL-ML caused no paw-lick responses and had a better anti-tumor effect in model mice. These results demonstrated that the ASL-ML using an active loading method by micelles-gradient can be utilized as sustained-release drug carriers for potential phlebitis prevention.

At last, a serum release model had also been developed which was more bio-relevance than PBS release model *in vitro* to predict the *in vivo* drug behavior (Silvander et al., 1998). The PBS release curves between ASL-ML (DPPG) and ASL-ML (HSPC) were similar. However, ASL-ML (DPPG) showed no success in controlling the leakage in *in vitro* serum release model. This may be because the lipids of HSPC had a higher PIT (51 °C) than DPPG (41 °C) which could be the reason for lower permeability of liposomal membrane. The PIT of DPPG is close to body temperature and equal to that of DPPC which may have proved to cause leakage. The enhanced drug retention exhibited by DPPG in PBS may come from the electrostatic interaction between drug and DPPG (Maurer-Spurej et al., 1999), which was however readily interfered by blood components such as charged proteins (Comiskey & Heath, 1990). These highlighted the importance of bio-relevance of *in vitro* release model for formulation screening.

Even the ASL-ML prepared by active loading method achieved a much high EE (2–4 times) than the conventional pre-loading method, ASL-ML (HSPC) possessed a relatively lower EE compared to ASL-ML (DPPG), which may result from the different fluidity of liposomal membrane. Therefore, removal of free drug before use is necessary for the current formulation and more conditions for drug loading needs to be optimized in the future. In addition, to make full use of the composite carriers, a responsive release of inner micelles in the tumor might also be necessary to improve the tumor penetration efficiency, except controlling drug release for optimized anti-tumor efficacy.

## Conclusions

The ML liposomes (micelles-contained liposomes) with high EE and improved drug retention have been constituted based on a novel active loading way using micelles as gradient for improved anti-tumor efficacy and potential phlebitis prevention during i.v. infusion. Results from TEM and fluorescent observation, influencing factors tests on EE and *in vitro* drug release study demonstrated the formation of ML-shell structure for ASL-ML without inter-carrier fusion or destruction, the location of drug mainly in inner micelles, and as well as the superiority of post-loading method to the pre-loading one, in which drug in micelles all shifted onto the bilayer membrane. The drug amphiphilicity and interaction of drug with micelles explained the mechanism for this method. The extended drug retention capacity of ML has been proved through pharmacokinetic study, drug irritancy assay, and *in vivo* efficacy experiments. In conclusion, ASL-ML prepared by active loading method can effectively load drug into micelles with expected structure and improve drug retention *in vitro* and *in vivo*. This study also provided a feasible fundamental research for multi-scale composite carriers, which can be utilized for smart tumor therapy with triggered release to meet the contradictory requirements for carrier size in circulation and tumor tissues.

## References

[CIT0001] Abraham SA, McKenzie C, Masin D, et al (2004). In vitro and in vivo characterization of doxorubicin and vincristine coencapsulated within liposomes through use of transition metal ion complexation and pH gradient loading. Clin Cancer Res 10:728–38. 14760096 10.1158/1078-0432.ccr-1131-03

[CIT0002] Baguley BC. (1990). The possible role of electron-transfer complexes in the antitumor anciton of amsacrine analogs. Biophys Chem 35:203–12. 2204443 10.1016/0301-4622(90)80009-v

[CIT0003] Baguley BC, Denny WA, Atwell GJ, et al (1984). Synthesis, antitumor activity, and DNA binding properties of a new derivative of amsacrine, N-5-dimethyl-9-[(2-methoxy-4-methylsulfonylamino)phenylamino]-4-acridineca rboxamide. Cancer Res 44:3245–51. 6547635

[CIT0004] Banno B, Ickenstein LM, Chiu GN, et al (2010). The functional roles of poly(ethylene glycol)-lipid and lysolipid in the drug retention and release from lysolipid-containing thermosensitive liposomes in vitro and in vivo. J Pharm Sci 99:2295–308. 19902527 10.1002/jps.21988

[CIT0005] Boman NL, Mayer LD, Cullis PR. (1993). Optimization of the retention properties of vincristine in liposomal systems. Biochim Biophys Acta. 1152:253–8. 8218326 10.1016/0005-2736(93)90256-y

[CIT0006] Cammas S, Matsumoto T, Okano T, et al (1997). Design of functional polymeric micelles as site-specific drug vehicles based on poly (α-hydroxy ethylene oxide-co-β-benzyl L-aspartate) block copolymers. Mater Sci Eng C 4:241–7.

[CIT0007] Comiskey SJ, Heath TD. (1990). Serum-induced leakage of negatively-charged liposomes at nanomolar lipid concentrations. Biochemistry 29:3626–31. 2340262 10.1021/bi00467a006

[CIT0008] Covey JM, Kohn KW, Kerrigan D, et al (1988). Topoisomerase II-mediated DNA damage produced by 4'-(9-acridinylamino)-methanesulfon-m-anisidide and related acridines in L1210 cells and isolated nuclei: relation to cytotoxicity. Cancer Res 48:860–5. 2827887

[CIT0009] Deng C, Jiang Y, Cheng R, et al (2012). Biodegradable polymeric micelles for targeted and controlled anticancer drug delivery: promises, progress and prospects. Nano Today 7:467–80.

[CIT0010] Edwards K, Johnsson M, Karlsson G, et al (1997). Effect of polyethyleneglycol-phospholipids on aggregate structure in preparations of small unilamellar liposomes. Biophysical J 73:258–66. 10.1016/S0006-3495(97)78066-4 PMC1180927 9199790

[CIT0011] Greish K, Sawa T, Fang J, et al (2004). SMA–doxorubicin, a new polymeric micellar drug for effective targeting to solid tumours. J Control Release 97:219–30. 15196749 10.1016/j.jconrel.2004.03.027

[CIT0012] Gubernator J. (2011). Active methods of drug loading into liposomes: recent strategies for stable drug entrapment and increased in vivo activity. Expert Opin Drug Deliv 8:565–80. 21492058 10.1517/17425247.2011.566552

[CIT0013] Kim JY, Lee H, Oh KS, et al (2013). Multilayer nanoparticles for sustained delivery of exenatide to treat type 2 diabetes mellitus. Biomaterials 34:8444–9. 23895999 10.1016/j.biomaterials.2013.07.040

[CIT0014] Lee RJ, Wang S, Turk MJ, et al (1998). The effects of pH and intraliposomal buffer strength on the rate of liposome content release and intracellular drug delivery. Biosci Rep 18:69–78. 9743475 10.1023/a:1020132226113

[CIT0015] Liu X, Sun W, Zhang B, et al (2013). Clarithromycin-loaded liposomes offering high drug loading and less irritation. Int J Pharm 443:318–27. 23337631 10.1016/j.ijpharm.2013.01.023

[CIT0016] Liu J, Zeng F, Allen C. (2005). Influence of serum protein on polycarbonate-based copolymer micelles as a delivery system for a hydrophobic anti-cancer agent. J Control Release 103:481–97. 15763628 10.1016/j.jconrel.2004.12.013

[CIT0017] Maurer-Spurej E, Wong KF, Maurer N, et al (1999). Factors influencing uptake and retention of amino-containing drugs in large unilamellar vesicles exhibiting transmembrane pH gradients. Biochim Biophys Acta 1416:1–10. 9889298 10.1016/s0005-2736(98)00204-1

[CIT0018] Mikhail AS, Allen C. (2009). Block copolymer micelles for delivery of cancer therapy: transport at the whole body, tissue and cellular levels. J Control Release 138:214–23. 19376167 10.1016/j.jconrel.2009.04.010

[CIT0019] Miller T, Breyer S, Van CG, et al (2013). Premature drug release of polymeric micelles and its effects on tumor targeting. Int J Pharm 445:117–24. 23384729 10.1016/j.ijpharm.2013.01.059

[CIT0020] Oh KS, Lee H, Kim JY, et al (2013). The multilayer nanoparticles formed by layer by layer approach for cancer-targeting therapy. J Control Release 165:9–15. 23103984 10.1016/j.jconrel.2012.10.013

[CIT0021] Permprasert J, Devahastin S. (2005). Evaluation of the effects of some additives and pH on surface tension of aqueous solutions using a drop-weight method. J Food Eng 70:219–26.

[CIT0022] Ravindra P. (2007). A critical review: surface and interfacial tension measurement by the drop weight method. Chem Eng Commun 195:889–924.

[CIT0023] Reimhult E. (2014). Nanoparticle-triggered release from lipid membrane vesicles. N Biotechnol 32:665–72. 25534673 10.1016/j.nbt.2014.12.002

[CIT0024] See E, Zhang W, Liu J, et al (2014). Physicochemical characterization of asulacrine towards the development of an anticancer liposomal formulation via active drug loading: stability, solubility, lipophilicity and ionization. Int J Pharm 473:528–35. 25079434 10.1016/j.ijpharm.2014.07.033

[CIT0025] Siiman LA, Lumeau J, Glebov LB. (2009). Porous nanoparticle supported lipid bilayers (protocells) as delivery vehicles. J Am Chem Soc 131:1354–5. 19173660 10.1021/ja808018y PMC2649781

[CIT0026] Silvander M, Johnsson M, Edwards K. (1998). Effects of PEG-lipids on permeability of phosphatidylcholine/cholesterol liposomes in buffer and in human serum. Chem Phys Lipids 97:15–26. 10081146 10.1016/s0009-3084(98)00088-7

[CIT0027] Sklarin NT, Wiernik PH, Grove WR, et al (1992). A phase II trial of CI-921 in advanced malignancies. Invest New Drugs 10:309–12. 1487405 10.1007/BF00944186

[CIT0028] Sunoqrot S, Bugno J, Lantvit D, et al (2014). Prolonged blood circulation and enhanced tumor accumulation of folate-targeted dendrimer-polymer hybrid nanoparticles. J Control Release 191:115–22. 24837188 10.1016/j.jconrel.2014.05.006 PMC4156894

[CIT0029] Wong C, Stylianopoulos T, Cui J, et al (2011). Multistage nanoparticle delivery system for deep penetration into tumor tissue. Proc National Academy Sci 108:2426–31. 10.1073/pnas.1018382108 PMC3038705 21245339

[CIT0030] Xin Y, Qi Q, Mao Z, et al (2017). PLGA nanoparticles introduction into mitoxantrone-loaded ultrasound-responsive liposomes: in vitro and in vivo investigations. Int J Pharm 528:47–54. 28559216 10.1016/j.ijpharm.2017.05.059

[CIT0031] Xu X, Khan MA, Burgess DJ. (2012). Predicting hydrophilic drug encapsulation inside unilamellar liposomes. Int J Pharm 423:410–8. 22207162 10.1016/j.ijpharm.2011.12.019

[CIT0032] Yuk SH, Oh KS, Koo H, et al (2011). Multi-core vesicle nanoparticles based on vesicle fusion for delivery of chemotherapic drugs. Biomaterials 32:7924. 21784512 10.1016/j.biomaterials.2011.07.017

[CIT0033] Zhang W, Falconer JR, Baguley BC, et al (2016). Improving drug retention in liposomes by aging with the aid of glucose. Int J Pharm 505:194–203. 27021465 10.1016/j.ijpharm.2016.03.044

[CIT0034] Zhang W, Wang G, Falconer JR, et al (2015a). Strategies to maximize liposomal drug loading for a poorly water-soluble anticancer drug. Pharm Res 32:1451–61. 25355460 10.1007/s11095-014-1551-8

[CIT0035] Zhang W, Wang G, See E, et al (2015b). Post-insertion of poloxamer 188 strengthened liposomal membrane and reduced drug irritancy and in vivo precipitation, superior to PEGylation. J Control Release 203:161–9. 25701612 10.1016/j.jconrel.2015.02.026

